# Industrial Applications of Terahertz Sensing: State of Play

**DOI:** 10.3390/s19194203

**Published:** 2019-09-27

**Authors:** Mira Naftaly, Nico Vieweg, Anselm Deninger

**Affiliations:** 1 National Physical Laboratory, Hampton Road, Teddington TW11 0LW, UK; 2 TOPTICA Photonics AG, Lochhamer Schlag 19, 82166 Gräfelfing, Germany; Nico.Vieweg@toptica.com (N.V.); Anselm.Deninger@toptica.com (A.D.)

**Keywords:** terahertz, industrial applications, polymers, paint coatings, pharmaceuticals, electronic circuits, petrochemicals, gas sensing, paper and wood

## Abstract

This paper is a survey of existing and upcoming industrial applications of terahertz technologies, comprising sections on polymers, paint and coatings, pharmaceuticals, electronics, petrochemicals, gas sensing, and paper and wood industries. Finally, an estimate of the market size and growth rates is given, as obtained from a comparison of market reports.

## 1. Introduction

**Figure 1:**
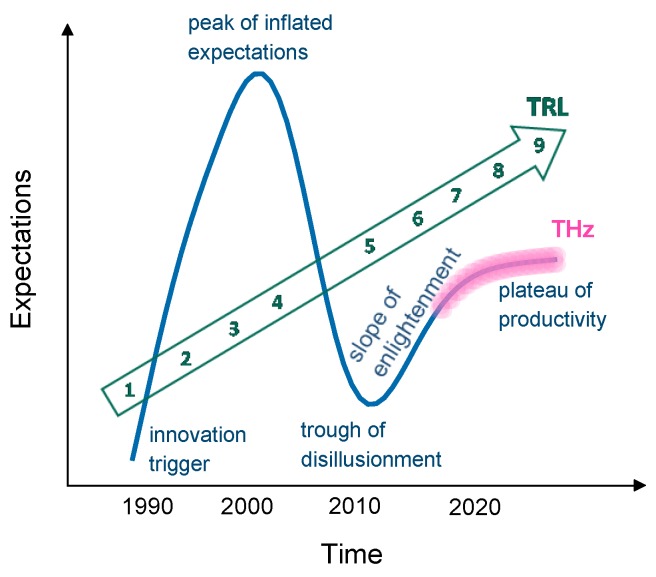
The Gartner Hype Cycle, TRL evolution, and the current envisaged position of THz technologies.

According to the now-famous Gartner hype cycle [1] (Figure 1), newly invented technologies experience a typical development path: from discovery to exaggerated expectations, followed by disillusionment, and on to the mature phase of steady productivity and growth. If mapped onto this scheme, THz sensing is currently on the threshold of this desirable “plateau of productivity”, at technology readiness level (TRL) 6–8.

The following publications well illustrate this development. Siegel [2] and Schmuttenmaer [3] (2003 and 2004, at the peak of hype) enthusiastically highlighted the possibilities and promise of THz technologies. Stöhr [4] (2009, beginning of disillusionment) highlighted the potential, yet with a note of caution, acknowledging the challenges of implementation. Armstrong [5] (2012, the trough) in a paper notably titled “The Truth about Terahertz” underscored the difficulties and barriers to THz applicability. Hochrein [6] (2015, slope of enlightenment) carried out market research, concluding that THz technologies will continue to grow in acceptance and usage, albeit primarily in specialised RD and measurement labs, and in a recent report (2019, approaching productivity) Frost and Sullivan included THz sensors as one of the top 50 emerging technologies [7].

At the time of writing, there are some existing industrial installations and several ongoing field trials of THz sensors; much development work is proceeding apace. Since THz sensing is an optical technique, it is natural that the great majority of envisaged industrial applications pertain to forms of non-destructive testing and condition monitoring. In particular, many deployments address thickness measurements of polymer structures and coatings. In this paper, we present a review of some of the more prominent and potentially promising demonstrators of THz sensing in industry. Section 2 is a brief description of the most widely used THz technologies. This is followed by sections focused on specific areas of application: polymers (Section 3), paint and coatings (Section 4), pharmaceuticals (Section 5), electronics (Section 6), petrochemicals (Section 7), gas sensing (Section 8), and paper and wood (Section 9). THz communications, which is a rapidly developing technology aimed at mass-market usage, lies outside the scope of this paper.

## 2. Instrumentation

To date, the vast majority of THz measurements have been performed using time-domain spectroscopy (TDS). Hundreds of papers have been published describing the technique and analysing its various aspects, including numerous excellent reviews [8,9,10]. Some of the salient points to bear in mind when using TDS are noted below. In order to utilise TDS data, it is necessary to understand certain aspects of system performance and the correct interpretation of the results.

**Figure 2:**
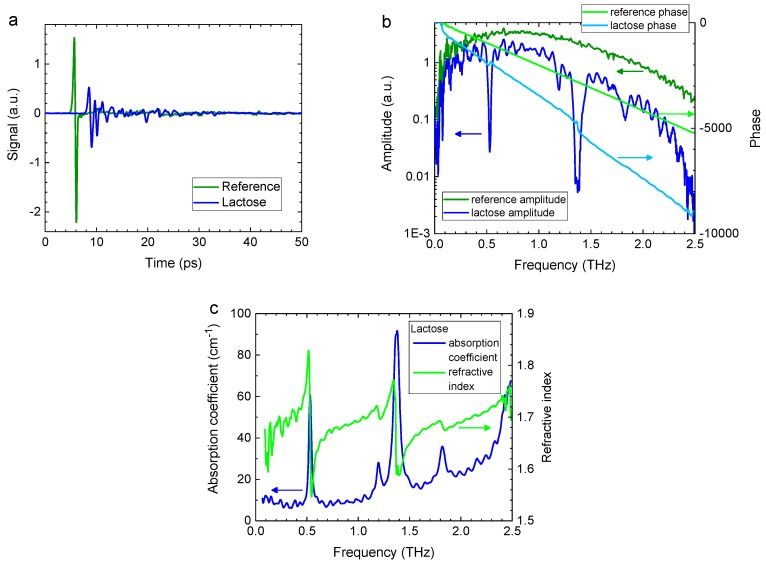
(**a**) Time-domain traces of a reference measurement and a lactose sample. (**b**) Spectral data (Fourier transform) of traces in (**a**). (**c**) Derived absorption coefficient and refractive index of lactose. Courtesy of M. Naftaly, NPL, UK.

- TDS is acquired in the time domain, i.e., it follows the time evolution of the signal (Figure 2a). Spectral data is derived by applying a Fourier transform (Figure 2b). As a result, there are two different approaches available for analysing the sensor data. Either time-domain or frequency-domain (i.e., spectral) data may be more useful for a particular application.
- TDS operates in a signal-probe configuration and uses short pulses (the THz pulse length is ~1 ps and the probe pulse length is <0.1 ps). As a consequence, there are no standing waves formed in the system itself or in the material/object measured. This simplifies data analysis.
- Because TDS uses short pulses and coherent detection, it provides an unambiguous measurement of the electromagnetic field amplitude and phase, which directly yield transmission loss and phase delay, and which in turn can be used to derive the absorption coefficient and refractive index of the material measured (Figure 2c).
- In most cases, TDS applications require a reference measurement, i.e., a data set recorded either without any sample or with reference material in place. However, once a reference data set is obtained for a specific system configuration, TDS instruments do not require repeated frequent calibration.
- The nominal operational bandwidth of a typical TDS system is 0.1–5 THz. However, this is reduced by transmission loss (according to a well-formulated dependence). The typical frequency resolution is ~5 GHz, down to 1 GHz is achievable. TDS is inherently broadband: each time-domain pulse trace yields a complete spectral data set.

Figure 2 illustrates the sequence of steps in a TDS measurement, as enumerated below; the sample is a pellet of lactose monohydrate.

1. A reference time-domain trace is recorded, followed (or preceded) by a measurement of the sample (Figure 2a).
2. A Fourier transform is applied to the time-domain data, yielding spectral data (Figure 2b). THz transmission loss in the sample is evident in the difference between the reference and sample spectra.
3. The THz optical parameters of the sample are calculated from the spectral data of reference and sample (Figure 2c) [11,12,13,14]. The refractive index is obtained from the phase data; whereas the absorption coefficient is derived from the amplitude data, taking into account the previously calculated refractive index.

There are two alternative techniques for implementing the requisite variable time delay between the THz and probe pulses: (1) mechanical stages [15,16,17], and (2) opto-electronic [18,19,20] techniques. The mechanical delay is the most widely used approach. Its advantages are excellent reproducibility, low jitter and low noise. Its main disadvantage is its comparatively slow measurement speed. Opto-electronic delay methods require two lasers with synchronized, yet slightly offset pulse repetition rates. Their main advantage is extremely fast data acquisition. This also leads to their main disadvantage which is a reduced SNR (signal-to-noise ratio), caused by shorter time intervals per data point. Indeed, the SNR decreases with faster acquisition rates.

Current-generation THz TDS instruments are compact, robust, flexible, comparable in price to other types of optical instrumentation (e.g., spectrometers, Raman systems), and suitable for installations in an industrial environment.

A frequency-domain spectrometer (FDS) is closely related to TDS in terms of physical processes, technological solutions, and measurement techniques [21,22,23]. It employs two continuous-wave lasers with offset wavelengths, dedicated semiconductor-based antennas, commonly referred to as “photomixers”, convert the beat signal into a monochromatic THz wave. Varying the wavelength offset between the two lasers tunes the THz frequency. Unlike TDS, FDS is continuous-wave, with the high temporal coherence giving rise to standing waves that must be accounted for in measurements. Similarly to TDS, FDS uses coherent detection, yielding information on both THz field amplitude and phase. The typical operating range is 0.05–3 THz [24]. The main advantages of FDS are a very high-frequency resolution (<5 MHz), the possibility of measuring at a user-selected, fixed frequency (or frequency range), and a comparatively low system cost.

**Figure 3:**
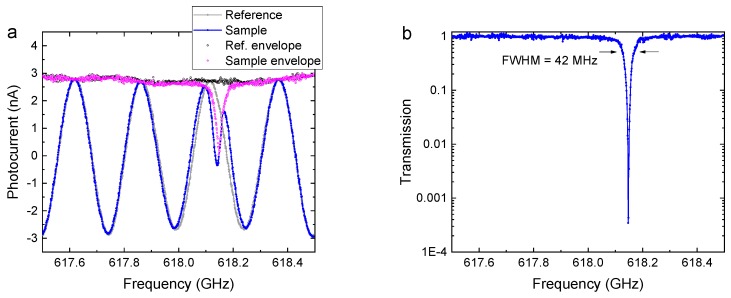
(**a**) Frequency-domain signals of a reference (grey) and sample (red) scans. The coherent detection scheme gives rise to phase “fringes”, which appear as an oscillating pattern when the THz frequency is varied. A Hilbert transform yields the envelope spectra of reference (black) and sample (pink). (**b**) The sample transmission spectrum (the square of the ratio of the sample and reference envelope data) reveals the whispering-gallery-mode resonance with an FWHM linewidth of 42 MHz. Courtesy of Dominik Vogt, University of Auckland, New Zealand.

Figure 3 illustrates the sequence of steps in an FDS measurement; the sample is a silicon sphere resonator with a whispering-gallery-mode (WGM) resonance [25].

1. Akin to TDS, a reference measurement is recorded, followed (or preceded) by a measurement of the sample (Figure 3a blue and grey). The phase-sensitive (coherent) detection scheme gives rise to phase “fringes”: the detected signal, i.e., the photocurrent measured in the receiver photomixer, oscillates between negative and positive values as the THz frequency is scanned. This effect is similar to scanning an interference pattern in frequency [26] and must not be confused with standing waves that arise due to multiple reflections of the beam. Note that the frequency step size in the example of Figure 3 is as small as 1 MHz, providing high-resolution spectral measurements.
2. In the first post-processing step, the envelope spectrum of the phase fringes is computed (Figure 3a purple and black). The most straightforward approach is a simple identification of the phase maxima and minima [26]; however, this method fails if the linewidth of the spectral feature is narrower than the fringe period. Phase and amplitude information for each frequency step is either obtained with phase modulation techniques or by applying a Hilbert transform to the raw data [27].
3. The envelope spectrum of the sample is divided by that of the reference measurement. The square of the resulting ratio produces the transmission spectrum (Figure 3b), where the ratio is squared because “transmission” refers to the intensity, whereas the envelope spectra of step (2) are proportional to the electric field of the terahertz wave.

In the example of Figure 3, the WGM resonance has a Q factor of 15,000 and an FWHM (full width half maximum) linewidth of 42 MHz. The line shape is clearly resolved with an FDS system, but such a narrow resonance would not be detectable at all with a TDS.

Similarly to optical measurements in the visible and NIR, it is possible to perform THz measurements by employing any suitable combination of a source and detector. The current range of technologies has been surveyed and assessed in several recent reviews [9,28,29,30]. The challenge lies in the inherently low intensity of THz sources and the low sensitivity of most THz detectors; nevertheless, in the low part of the THz range (<1 THz) useful electronic sources and detectors are now available. Multipixel detector arrays and video-rate THz cameras are also available from several manufacturers [31,32,33,34] and may find industrial use in the future.

The following sections offer a review of current industrial applications of THz technologies, describing demonstrated and deployed systems. Some of these applications can in principle be realized using different types of THz instrumentation (for example TDS, FDS, or single-frequency electronic emitters/detectors). However, the remit of this paper is to present existing implementations.

## 3. Polymers

Polymers are transparent or semi-transparent for terahertz waves and thus among the most important materials for implementations of THz technology [35]. A large body of literature exists reporting THz measurements of polymer properties and behavior. The fields of application range from the characterization of macroscopic and morphological properties [35,36] to the non-destructive testing of polymers and polymer products [37,38]. Polymers are also used as materials for THz optics [39,40] and as a material for THz emitters and detectors [41]. In particular, two of the most widely used polymers, polypropylene and polyethylene, are among the most transparent materials to THz waves. The materials covered in this section include pure polymers as well as polymer foams, polymer composites and adhesives.

### 3.1. Polymers and Polymer Components

One of the most important industry market drivers for THz technology is currently the field of non-destructive testing of plastics and plastic products. Plastics have become an indispensable part of our everyday lives. The global market for plastics was 263 million tons in 2016 and the projected growth is 3–5% per year (https://www.ceresana.com; https://www.farbeundlack.de).

In line with the market size and growth, there is an enormous demand for test equipment, both to ensure acceptable product quality and to reduce material usage and wastage. With material savings in production of approx. 1%, even a test instrument of about €100 k will amortize itself within 1–2 years. Currently, established and cost-effective nondestructive testing technologies include X-ray scanners, ultrasonic sensors and microwave transceivers. However, THz technology has now reached the cost levels, size, robustness, measurement speeds and discrimination quality of previous gold standards [42,43,44].

THz technology offers a number of advantages enabling it to partially replace other established techniques in applications such as inline measurements in plastic extrusion processes [43]. Moreover, it wins new fields of application such as the inspection of multi-layer paint coatings in the automotive industry as described in Section 4. Unlike X-ray scanners, THz systems do not emit ionizing radiation and are considered biologically innocuous, thus saving costs entailed in extensive radiation protection measures, safety officers and compliance documentation. In addition to being nondestructive, THz systems operate contact-free and do not require a contact medium, as do ultrasonic sensors. Crucially, THz waves can penetrate polymers, even in cases where the material is optically opaque, ultrasound is strongly damped, and near-infrared waves are scattered. Compared to microwaves, THz waves achieve higher spatial resolution, so that submillimeter-sized defects can be detected or micrometer-thin polymer coatings can be analyzed.

**Figure 4:**
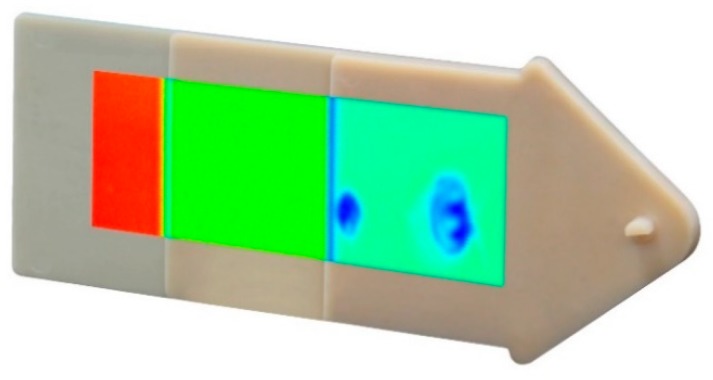
Plastic step wedge made of polyamide. Air pockets are made visible using THz TDS. Adapted from [15], courtesy of TOPTICA Photonics AG.

Due to their good optical penetration through most polymers, THz systems are particularly suitable for the inspection of plastic components and the detection of defects [45,46]. Figure 4 shows a step wedge made of polyamide. Two internal air pockets approx. 5 mm and 10 mm in size are invisible to the naked eye or an optical system but are revealed using THz imaging. The sample was raster scanned over an area of 100 mm × 40 mm using a commercial TDS instrument [15,47]. The thickness of the individual sections was 1 mm, 2 mm, and 4 mm, red and blue colors indicate high and low transmission respectively. The air bubbles appear “blue” because THz waves are strongly scattered at the air-polymer interfaces, reducing transmission.

**Figure 5:**
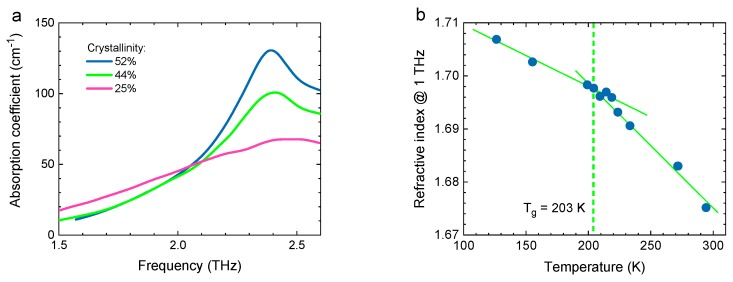
(**a**) Absorption spectrum of three samples of polybuthylene terephathalate (PBT) with different degrees of crystallinity. (**b**) Temperature-dependent refractive index of polyoxymethyllene (POM). The glass transition temperature can be determined from the intersection of the two linear slopes. Figures adapted from [35], courtesy of S. Wietzke, Continental AG.

THz techniques can reveal not only voids but also thermal or mechanical stress, deformations and ageing processes in polymer materials. The effects of these processes can be seen, for example, by observing changes in the degree of crystallinity [48,49] or changes in the glass transition temperature [50]. Figure 5a shows the THz absorption coefficient of polybutylene terephtalate (PBT) with different degrees of crystallinity. As crystallinity increases, the absorption peak at 2.4 THz becomes more pronounced. Figure 5b shows the THz refractive index of polyoxymethylene (POM) as a function of temperature. The glass transition temperature can be determined from the intersection of the two straight dashed lines. In addition to static polymer properties, dynamic behavior, such as crystallization [49] and polymerization [38], can also be observed and tracked.

### 3.2. Composite Materials

**Figure 6:**
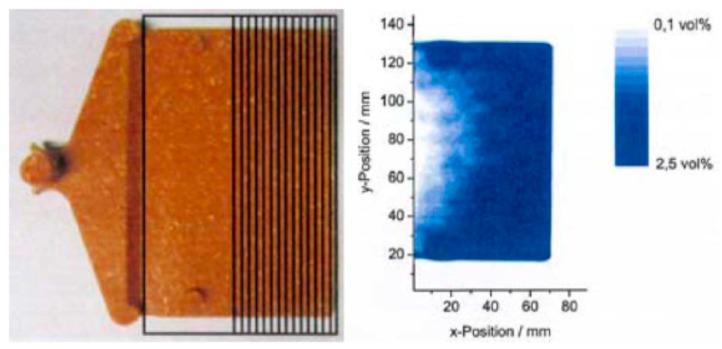
Photograph (left) and THz transmission image (right) of a wood/plastic composite material after immersion in water. From [35], courtesy of S. Wietzke, Continental AG.

Most plastic components are not made of pure polymers but of composite materials. During the compounding process, a polymer material is adapted to the specific application by incorporating additives. These include, for example, filling with chalk, reinforcing with glass fibers or dyeing with colorants. For the plastics industry, it is important to verify the additive content [51], the homogeneous distribution of fillers, i.e., the degree of dispersion [52], the moisture content [53], as well as the orientation of fibers [54], particles [55] and molecular chains [56]. The properties of a composite material can be determined by measuring its THz refractive index and absorption coefficient [35]. As an example of moisture-contrast imaging, Figure 6 shows a photograph and the corresponding THz TDS transmission image of a wood-plastic composite containing 60% by weight of wood fibers, that had been immersed in water for four days. The frequency interval between 0.4 and 0.5 THz was used to generate the image, as a compromise between poor spatial resolution at lower frequencies and strong absorption at higher frequencies. The presence of water is revealed in areas of reduced THz transmission because THz waves are strongly absorbed by water.

### 3.3. Polymer Foams

With a global market volume of 19.1 million tons in 2013, polymer foams are one of the most important classes of polymers [57]. They have excellent thermal and mechanical properties at low weight and are used to increase energy efficiency in the construction, automotive, aerospace and rotor blade industries. In the past, testing of these materials was time-consuming, partially destructive and only possible offline. While polymer foams strongly damp ultrasound, they have good transparency to THz waves. Today, THz systems are used in the extrusion industry to determine the wall thickness of polymer foam pipes [43].

**Figure 7:**
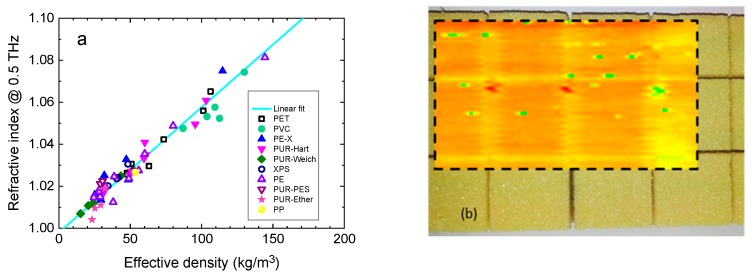
(**a**) Relation between refractive index and effective density for various polymer foams. Adapted from [52], courtesy of M. Mayr, Sűddeutsches Kunststoffzentrum. (**b**) Photograph and THz transmission image (100 mm × 60 mm) of a 20 mm-thick piece polyvinyl chloride foam, as used in rotor blades. Courtesy of TOPTICA Photonics AG.

The cellular structure and the effective density of the foam are decisive quality parameters that can be determined by THz sensing inline during the production process [58]. The foam cells act as scattering centers for THz waves so that the cell size can be determined by investigating the THz loss coefficient (which combines contributions from absorption and scattering). In addition, there is a linear relationship between the bulk density and the effective THz refractive index (because the effective refractive index is proportional to the thickness of material in the beam path). Figure 7a shows an example of this relationship for a frequency of 0.5 THz as determined by TDS. The refractive index increases linearly with the effective density.

In addition, defects in the foam structure that can influence its mechanical properties can be detected with THz imaging techniques. Figure 7b shows a 20 mm thick polyvinyl chloride foam component as used in a rotor blade. In the overlaid 100 mm × 60 mm THz transmission image the defects in the sample are revealed as green and red dots.

### 3.4. Adhesives

**Figure 8:**
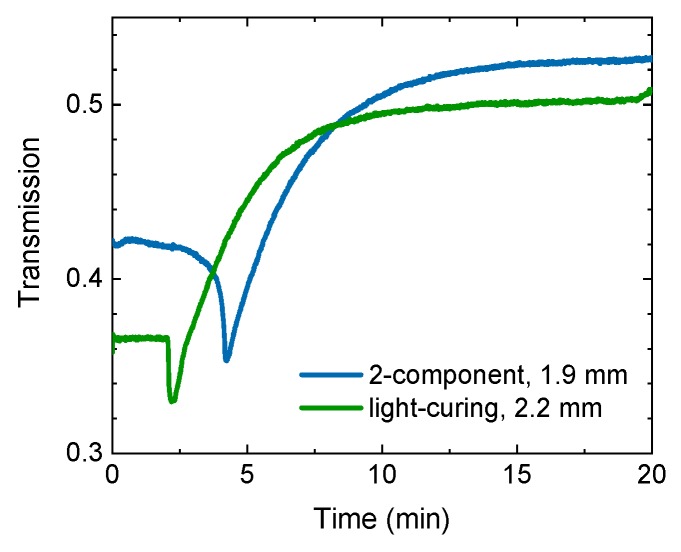
THz transmission through a 2-component and a light-curing adhesive during the curing process. Courtesy of TOPTICA Photonics AG.

Adhesives are another important class of polymers widely used in manufacturing and construction processes. However, non-destructive testing of adhesive joints is still a challenge. It has been demonstrated that THz instruments can be used to determine the thickness of adhesive joints and the distribution of adhesives between plastic components [59]. As with polymers, moisture can also be detected in an adhesive [60]. In addition, the curing behavior of adhesives can be investigated [61,62]. In the case of a 2-component glue, polymerization is initiated when two monomers, a binder and hardener, are mixed together; whereas in a light-curing glue the polymerization reaction is driven by absorption of light of appropriate wavelengths. Figure 8 shows the variation of transmission of pulsed THz radiation through a 2-component and a UV-curing adhesive during the curing process. The intensity of the THz pulses is detected with a Schottky diode [63]. The drops in intensity after 2 min and 4 min respectively mark the beginning of the polymerization. In both cases the polymerization reaction is exothermal: the adhesive initially heats up and softens, absorbing the THz waves more strongly. During curing, the refractive index increases and the absorption decreases. The increase of the effective refractive index is linked to the increase in polymer concentration and optical density. The decrease in the effective absorption coefficient correlates to the decrease in the concentration of the monomer because the monomer material has higher absorption than the polymer form [61].

## 4. Paint and Coatings

High-tech surfaces in the automotive or aircraft industry must withstand harsh environmental conditions such as heat, frost, and water, and impact from e.g. sand and stone-chips. In order to protect these surfaces and to render them visually attractive, they are encased in multilayer functional coatings and lacquers in an elaborate process. In the automotive sector, for example, typically three or four different layers are applied to the car body [64].

In order to ensure that the layers remain functional throughout the lifetime of the product and to minimize expenditure of the coating materials, the coating thickness must be carefully monitored. The global market for thickness measurement instruments registers a continuous substantial growth, which is due to various factors, including strict government regulations to ensure quality, growing safety concerns among buyers, the drive for cost optimization, and a rising trend for technology miniaturization (www.marketsandmarkets.com). The film thickness measurement market is expected to reach $520 M by 2023 at a CAGR of 4.9% (www.IGOS.de). An overview of established thickness measurements techniques can be found in [44].

Complex multi-layer coatings push established measurement technologies to their limits. Moreover, some of the conventional techniques, such as magnetic gauges or eddy-current measurements, fail in the case of non-metallic substrates, such as glass-fiber reinforced plastics. Until recently it has only been possible to test the paint on these substrates using destructive methods in which the surfaces are destroyed with a wedge cut [44].

**Figure 9:**
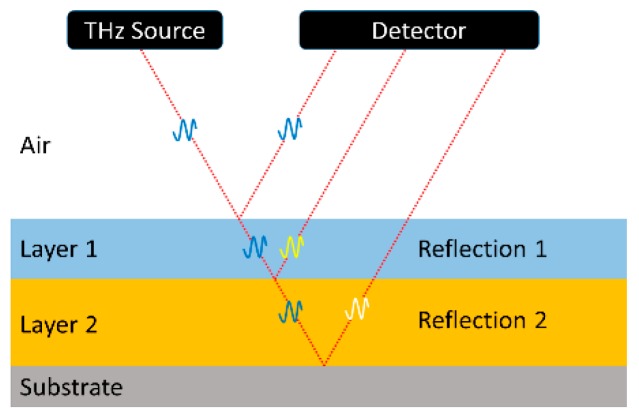
Schematic drawing of a substrate with two coating layers and the reflected THz beam path.

THz TDS provides a nondestructive solution for monitoring multilayer coatings on any substrate, enabling each layer thickness to be measured individually [63,64,65,66]. Today, such systems are already deployed in industrial installations [44,65,66,67,68,69,70,71,72,73,74]. Figure 9 depicts a substrate with two coating layers, together with the path of a reflected THz beam. Akin to thickness measurements with ultrasound, a THz beam is partially reflected from each of the interfaces. By recording the time-of-flight echo signals, the thickness of each individual layer can be resolved. By using a beam spot of less than 2 mm diameter and a spectrally broad THz pulse, it is possible to measure layer thicknesses down to 5–10 μm [70,75]. A spectrally broad THz pulse is necessary because the duration of a pulse is inversely related to its spectral width. Since the measurement technique relies on distinguishing closely spaced echo signals, it requires a short (i.e., broadband) THz pulse.

**Figure 10:**
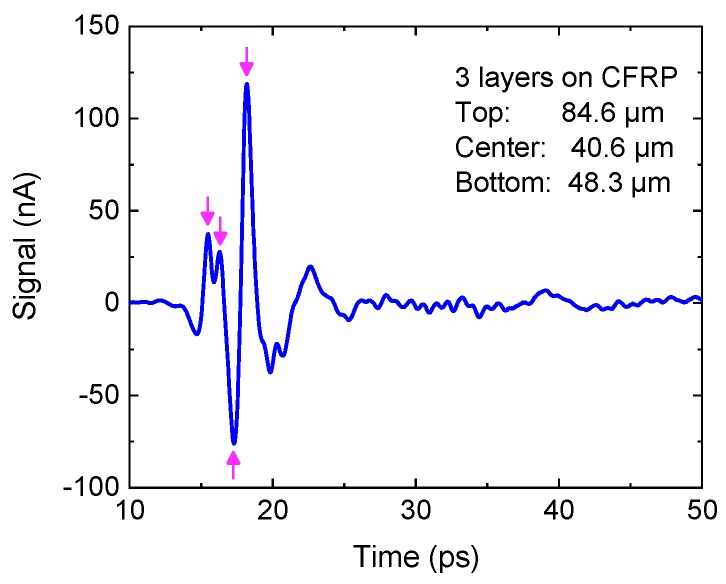
A THz pulse trace reflected from a three-layer coating on a carbon-fiber-reinforced plastic substrate. The echoes from the four interfaces are marked with arrows. Thicknesses of the individual layers are indicated. Courtesy of TOPTICA Photonics AG.

**Figure 11:**
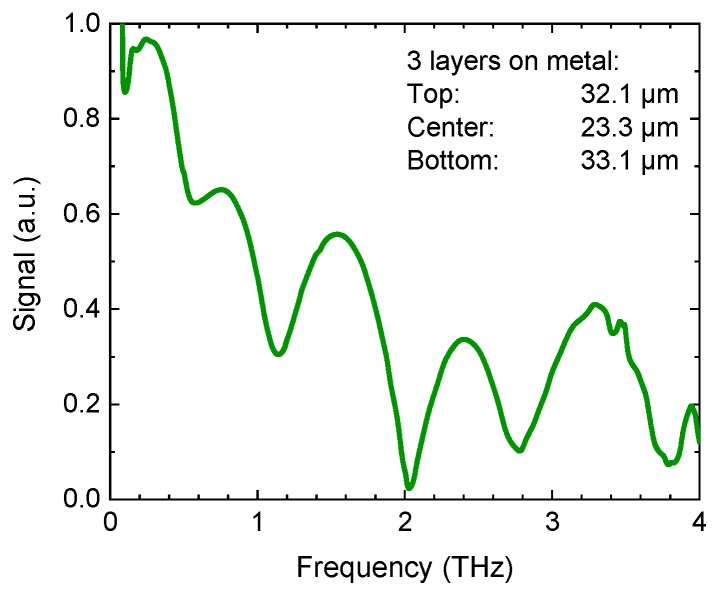
The frequency-domain representation of a THz pulse reflected from a three-layer coating on metal. Thicknesses of the individual layers are indicated. The layers give rise to interference signatures in the transfer function. Courtesy of Helmut Fischer GmbH.

Figure 10 shows the result of a THz reflection measurement of a three-layer coating with thicknesses of 84.6 µm (top), 40.6 µm (center), and 48.3 µm (bottom) on a carbon-fiber-reinforced plastic substrate (CFRP). The arrows represent the echo pulses from the four interfaces, which are in close proximity but can still be separated in the time-domain trace. For even thinner layers, the echoes begin to overlap and can no longer be resolved visually, and sophisticated evaluation algorithms are required to calculate the layer thickness from the raw data [75,76,77]. In particular, algorithms involving mathematical models of the transfer function, as well as “stochastic differential evolution” fitting routines [75], allow assessing layers on the order of 10–20 µm in a multilayer stack. Some of the analysis routines make use of fitting algorithms in the frequency-domain, rather than the time-domain since these thin layers manifest themselves as interference signatures in the transfer function (Figure 11).

THz systems have several advantages compared with other technologies: they operate contact-free, whereas ultrasonic and eddy current sensors require physical contact with the sample. THz instruments can, therefore, measure freshly deposited (“wet”) coatings. They can also provide reproducibility below 1% [44]. In comparison, the reproducibility with magnetic induction sensors is approx. 10%, which is insufficient for accurate measurements. The calibration effort for ultrasonic systems is high but is negligible for THz instruments [67]. Some coatings are opaque to optical and infrared beams, whereas THz waves penetrate most types of paints and varnishes [44,75].

In order for THz measurement systems to reliably measure thin layers in an industrial environment, solutions are needed to integrate this technology into production processes. On the one hand, a high signal-to-noise ratio and a large THz bandwidth are required in order to resolve layers of a few micrometer thicknesses at high measurement speeds [78,79]. On the other hand, the system must be cost-effective and robust [80].

In addition, clever algorithms are needed to calculate layer thickness information from the THz data in real-time [20] and under industrial conditions. Vibrations of automotive parts can interfere with the THz signal and impede or obstruct thickness measurements. However, such vibrations can be measured using optical methods and the THz pulse traces can be corrected in software [78]. Water vapor in the atmosphere has multiple absorption lines at THz frequencies (see Section 8) [81], leading to artifacts in the THz pulse that can mask relevant thickness information [42]. These artifacts can be minimized either numerically [82,83] or by purging the THz path with dry air or other gases [81].

**Figure 12:**
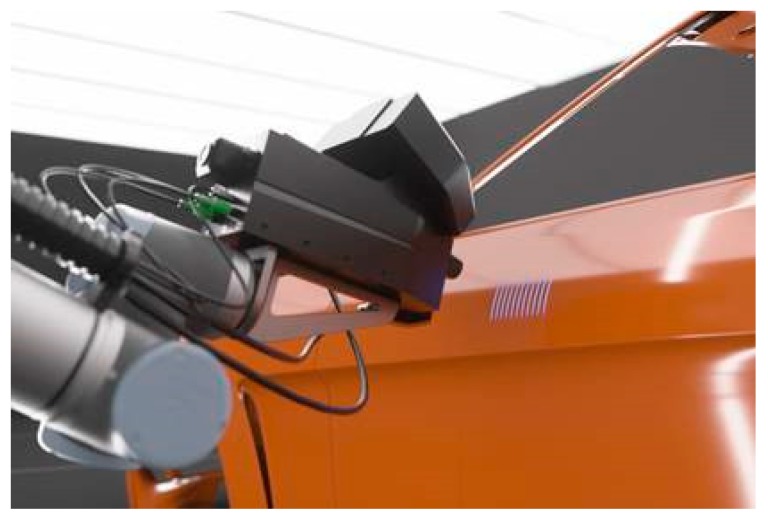
Implementation of a THz TDS in an industrial setting. Courtesy of Helmut Fischer GmbH.

Figure 12 shows an example of an implementation of a THz TDS system in the automotive industry [44]. The THz reflection head is aligned to the car body with the help of a robot arm. Optical sensors for distance and orientation provide closed-loop positioning of the THz head relative to the car body and are also used for the correction of vibrations.

## 5. Pharmaceuticals

The pharmaceutical industry is a particularly promising target area for THz applications, both in RD and for purposes of condition monitoring and non-destructive testing. There are three main reasons for this. First, pharmaceutical materials are (semi-) transparent at THz frequencies and often possess characteristic spectral signatures, whereas they are opaque in the visible and near-infrared. Second, high-precision quality monitoring is of immense importance in pharmaceutical production, because the efficacy and safety of a medicinal product are contingent on accurate control of both its chemical composition and its microstructure and mechanical properties. Thirdly, pharmaceuticals are high-value products where increased product consistency and reduced wastage bring significant cost benefits, therefore justifying investment in relatively expensive technologies such as THz sensing that improve the effectiveness of quality monitoring.

**Figure 13:**
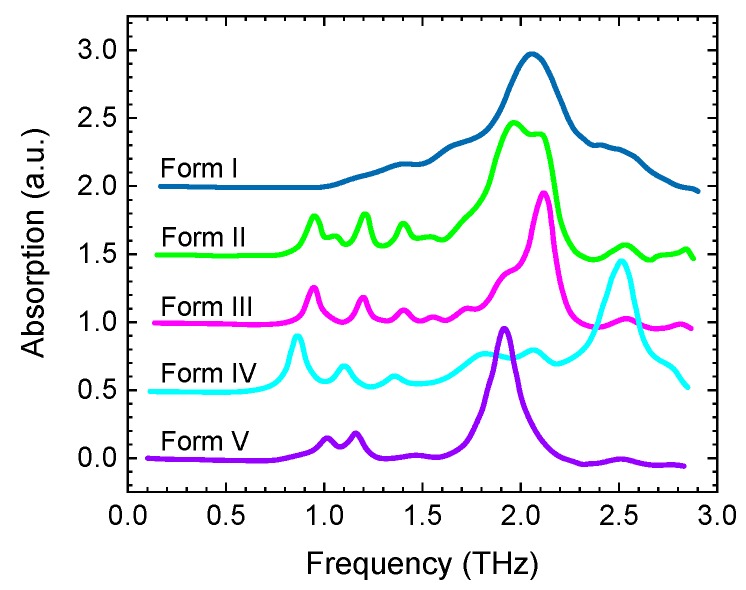
Terahertz absorption spectra of the five polymorphic forms of sulfathiazole (vertically offset and normalized for clarity). Adapted from [84].

From the early days of THz spectroscopy, it was realised that it can reveal variations in molecular configurations such as polymorphs, chirality, cocrystals, and crystalline or amorphous state (Figure 13) [84] (and references therein). Since then a large number of such studies have been published e.g., [85,86,87,88,89,90], and uses of THz spectroscopy have been continuously growing in pharmaceutical and biochemical RD. However, detailed spectroscopic measurements require precise control of experimental conditions and specific forms of sample preparation, and are neither suitable nor desirable for direct industrial applications such as in-line production monitoring or near-line quality inspection of finished products.

Industrial applications of THz sensing in the pharmaceutical industry have focused on two main areas: inspection of tablet and capsule coatings; and monitoring of tablet porosity and pore size. Pharmaceutical tablets and capsules are the most popular and widespread form of administering drugs to patients due to their cost-effectiveness, ease of use and patient compliance, with approximately 60% of medicines being delivered as oral solid dosage forms. Both tablets and capsules are formed by blending the active pharmaceutical ingredient (API) and excipient. Tablets are compacted into a solid pellet, and often coated with a specialized protective layer. In contrast, capsules consist of uncompacted power which is encased in a specialised shell. The functional performance of tablets and capsules is strongly dependent on the physical and structural properties of both the coating and the internal blend. These are the target application areas of THz sensing, for which it was shown to be particularly suitable.

Process analytical technology (PAT) is “a system for designing, analysing, and controlling the manufacturing of pharmaceutical compounds through timely measurements (i.e., during processing) of critical quality and performance attributes of raw and in-process materials and processes, with the goal of ensuring final product quality” (Federal Drug Administration Guidance). Demonstrating the feasibility and utility of THz measurements as a valid PAT tool is therefore crucial for its acceptance and uptake by the industry.

### 5.1. Tablet Coating Inspection

Pharmaceutical capsules consist of an enclosing soluble shell containing a loose powder blend that commonly combines an active ingredient (API) and an excipient. Pharmaceutical tablets are formed by compressing the powder blend into a solid pellet to which a coating is commonly applied, in part to mask their taste and odour. The most important functions of tablet and capsule coatings—which determine their performance in drug delivery—are twofold: to preserve drug functionality while in storage, and to facilitate timely drug dissolution (which may involve rapid or timed-release or release at the correct location in the gastrointestinal tract). The performance of coatings can be affected by their thickness, structural uniformity, and defects. Whereas inspection of such coatings is very challenging, especially with respect to coating thickness.

**Figure 14:**
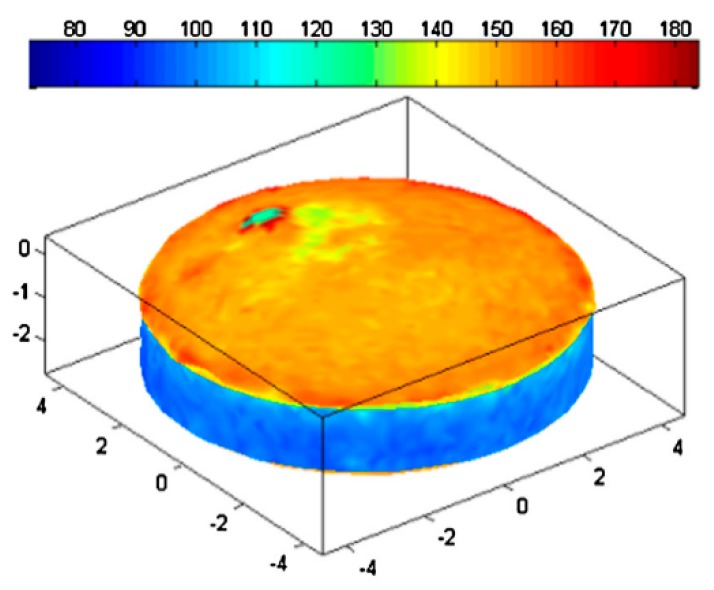
3D images of coating thickness of a biconvex tablet. The colour scale is in μm, the X-Y-Z scale is in mm. Reprinted with permission from [92] © Elsevier.

Reflection THz TDS offers an obviously suitable tool for monitoring coating thickness because the time delay between the two reflection signals from the surface and from the coating-core interface provides a direct measure of the coating layer thickness (see also Section 4). This was first demonstrated for tablets by Fitzgerald et al. in 2005 [91]. Since then much work has been done in this area, for both tablet and capsule coatings; and a comprehensive review was published by Haaser et al. in 2013 (Figure 14) [92] (and references therein), while more recent work has focused on specific industry-relevant aspects. A dedicated fully automated commercial THz instrument for tablet coating inspection using a 6-axis robotic arm to map the tablet surface was also developed [93].

There are two main challenges. The spatial resolution is limited by the wavelength of THz radiation to about 100 μm at best. More important, reflection measurements—the standard approach to thickness gauging—are made difficult by the curved surfaces of tablets and capsules, whose curvature is of the same order as the THz wavelength.

**Figure 15:**
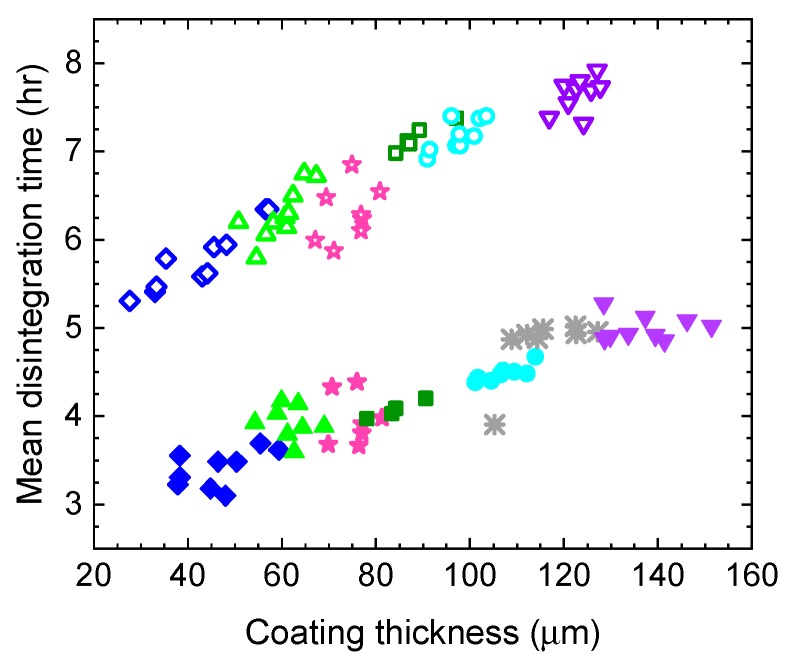
Relationship between tablet coating thickness and their MDT (mean dissolution time) for tablets produced by two manufacturing processes: lab-scale (using laboratory equipment, solid symbols) and pilot-scale (using industrial equipment; hollow symbols). Adapted from [94].

Several studies have demonstrated the utility of such measurements for monitoring tablet dissolution performance by confirming the dependence of mean dissolution time (MDT) of tablets on their coating thickness (Figure 15) e.g., [92,94] (and references therein). MDT is the standard reference technique used by the pharmaceutical industry to test the dissolution performance of solid dosage medicines.

Recent work in this area has focused on demonstrating the feasibility and utility of industrial applications. Examples include the use of THz sensing as a PAT tool for evaluating coatings deposited under different manufacturing conditions [95], observing the effect of processing conditions on inter-tablet variations in coating thickness [96], and development of a thickness measurement technique for two-layer coatings on capsules [97].

### 5.2. Monitoring Porosity and Pore Size

Timely dissolution of a pharmaceutical tablet is one of the most important aspects of drug delivery. Tablet disintegration is controlled by its chemical and mechanical properties. During the compression of a tablet, particles are consolidated to form interparticulate bonds and pores. Tablets are commonly manufactured by uniaxial compaction of powder confined radially in a rigid die. This directional compaction results in anisotropic mechanical properties and pore structure, where pores are elongated and predominantly aligned in the plane of the tablet. The pores in a tablet—their size and connectivity—directly affect the rate at which the physiological fluids enter the tablet, leading to swelling of the particles and eventually causing the break-up of the compact into smaller agglomerates. The size of the disintegrated particles then drives the dissolution rate of the drug. These mechanisms are strongly interconnected, as the swelling of particles dynamically changes the internal pore structure which influences the liquid imbibition process. Consequently, the tablet microstructure and the disintegration process play a pivotal role in product performance, and the performance of a tablet can be predicted and optimized by understanding the relationship between the dissolution rate and the granularity and porosity of the tablet material. Despite the widely recognized importance of monitoring the tablet porosity during the manufacturing process, there are no continuous in-line non-destructive techniques for achieving this. THz technology is a highly promising solution to this urgent problem due to its ability to directly measure porosity and its sensitivity to pore size. A body of work has been carried out aimed at developing it into an industrial tool.

Porosity has a dual effect on THz transmission properties of a material. The effective refractive index decreases roughly linearly with porosity because the effective interaction length with the material shortens (there is less substance in the beam path). The ability of THz TDS to provide a direct measurement of the effective refractive index, therefore, makes it highly suitable for monitoring and characterising porosity. In contrast to refractive index, loss tends to rise with porosity. This is because transmission loss combines contributions from absorption and scattering. Whereas the effective absorption of porous material declines with shorter interaction length, scattering rises strongly due to higher pore density and/or larger size of pores. Since pharmaceutical materials generally have moderate THz absorption, this tends to result in the combined loss that increases with porosity. However, the relationship between loss and porosity is often complicated and strongly dependent on material absorption and the pore geometry and distribution.

**Figure 16:**
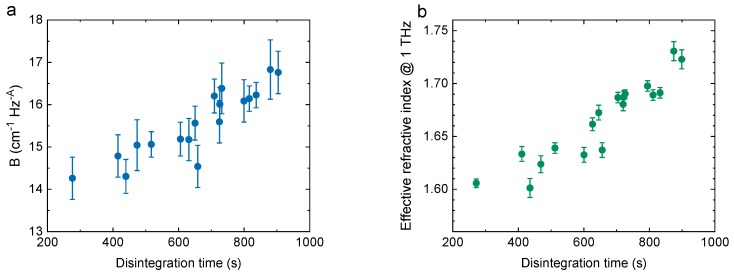
(**a**) Relationship between the loss parameter *B* and tablet disintegration time. The frequency-dependent scattering loss is described by *α*_eff_ = *B f ^A^* (where *A* ≈ 3.3), therefore the loss parameter *B* reflects the strength of transmission loss. (**b**) Relationship between effective refractive index and disintegration time. Adapted from [101].

The first demonstration of porosity measurements in pharmaceutical tablets using THz TDS was described by Ervasti et al. in 2012 [98], with many studies following, summarised by Markl et al. in [99] (and references therein). Since tablet dissolution depends on pore configuration, distribution and connectivity, as well as their size and size variation, recent work has aimed to develop techniques for accurately evaluating these properties in addition to calculating mean porosity [100,101,102,103,104]. Of particular interest is the relationship between THz transmission properties and tablet dissolution (Figure 16) [101], because dissolution time is a critical parameter in drug delivery. The behavior of powder during the manufacturing process and the effects of density variations in the final product have also been studied [105].

## 6. Electronics

From the early days of THz technologies, they were envisaged as an inspection tool for the electronics industry. This was primarily because electronic properties of semiconductor materials such as carrier concentration and mobility determine their dielectric properties at THz frequencies, allowing non-destructive, non-contact measurements. Hermann et al. [106] described an early demonstration using THz TDS to map areas of the differing carrier concentrations in silicon wafers, achieving a spatial resolution of ~1 mm. Moreover, optical inspection techniques are often unsuitable for electronic circuits and devices due to the opacity of the substrates and/or packaging.

Subsequent work in this field has mostly focused on three areas: inspection of electronic circuits; inspection of solar cells; and conductivity measurements/mapping of thin films, particularly as applied to graphene.

### 6.1. Electronic Circuits

The spatial resolution of far-field THz imaging is of the order of ~1 mm, being limited by the long wavelengths, and is therefore insufficient for detailed inspection of electronic circuits. Hence much work has been devoted to developing THz imaging techniques capable of delivering a sub-wavelength resolution.

In a series of publications Yamashita et al. described the development and progressive refinement of a “laser terahertz-emission microscope” (LTEM), achieving a spatial resolution of a few μm [107,108,109,110,111]. The LTEM operates by raster-scanning the circuit with a focused beam from a femtosecond laser. When the laser is incident on photoconductive structures, it generates a transient photocurrent, causing a THz pulse to be emitted. Structures capable of emitting THz, and therefore suitable to be examined by the LTEM, include photoconductive switches with an external bias voltage (i.e., Auston-type switches), unbiased interfaces carrying electric fields (e.g., p-n junctions, Schottky contacts), and some semiconductor surfaces. As with all photoconductive THz emitters, the THz field amplitude is proportional to the local electric field. The emitted THz radiation is collected and analysed, producing an image of the circuit.

**Figure 17:**
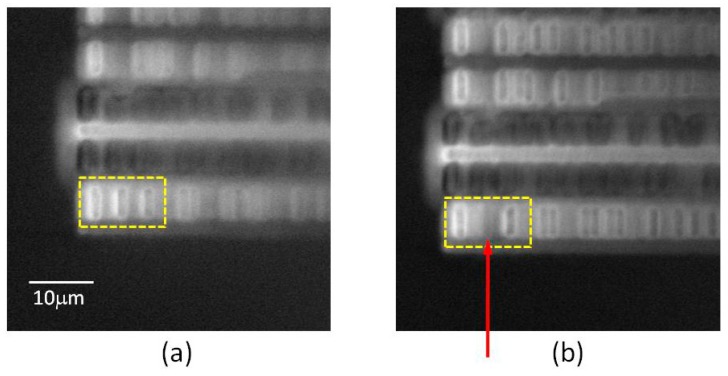
LTEM images of a functioning circuit (**a**) and a defective one (**b**). The arrow shows the location of the broken contact. Brighter areas indicate stronger THz emission. Reprinted with permission from [112] © The Optical Society.

Following the initial demonstration of LTEM imaging [107], its resolution was improved to better than 3 μm [108]. Directing attention to p-n junctions, defective transistor circuits were distinguished from correctly functioning ones [109,110]. Then imaging from the rear side of the circuit was shown, allowing non-contact inspection where interconnect structures prevent optical access from the front [111]. To facilitate characterization, laser-activated THz waveforms emitted from p-n junctions were studied in detail [112]. Figure 17 shows the ability of the LTEM to detect defects (broken contacts) in a circuit [112]. The capabilities of the LTEM were further extended and its spatial resolution was increased to 0.6 μm by incorporating near-field techniques [113]. A similar approach was applied to time-resolved transmission imaging, demonstrating measurements of carrier lifetimes and mobilities with a spatial resolution of 60 µm [114].

In order to image individual microelectronic components, a spatial resolution of <100 nm is necessary. Huber et al. employed scattering near-field optical microscopy (SNOM) using a 2.54 THz gas laser (methanol), achieving resolution of about 40 nm [115]. THz-SNOM imaging revealed individual transistors, identified the materials in a device, and was able to measure carrier concentrations. Although such systems are still too complex for industrial deployments, they can provide insights into device structure and performance.

Time-domain reflectometry (TDR) is a widely used non-destructive technique for detecting faults and discontinuities in electronic circuits, a task traditionally addressed by using a pulse generator and an oscilloscope. Nagel et al. changed the conventional method to a non-contact one by employing a TDS system and custom-built probes with a tip radius of 0.2 μm [116]. Their THz-TDR instrument was capable of locating discontinuities in electronic structures with a spatial resolution of 0.55 μm. Commercial THz-TDR instruments are currently deployed in the semiconductor industry.

More recent work focused on applying THz measurements for non-destructive inspection of packaged power electronic devices, obtaining images of their spatial features through the encapsulating coatings [117]. The approach adopted was similar to that used for measuring layers of car paint (Section 4) and coatings on pharmaceutical tablets (Section 5), achieving a spatial resolution of ~0.5 mm.

**Figure 18:**
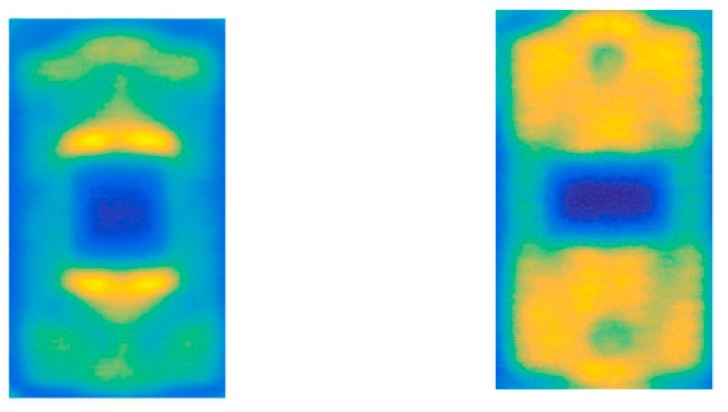
Transmission images of an authentic (right) Intel chip and a counterfeit one (left). Reprinted with permission from [118] © Elsevier.

Ahi et al. employed TDS combined with a range of measurement and analysis techniques to test a variety of attributes for quality control and authentication of packaged integrated circuits [118]. The aspects tested included: the presence of unexpected materials in counterfeit devices, blacktopping layers (used by counterfeiters to hide the original label and over-print a false one), shape and dimensions of hidden structures, sanded and contaminated devices, differences between internal structures of counterfeit and authentic devices, such as misshapen die-frames and bond-wires. The measurement techniques used were: transmission spectroscopy for material characterization, time-of-flight tomography, and reflection and transmission imaging. The image resolution was increased to ~ 0.1 mm by applying a deconvolution technique and taking into account the point spread function of the emitter. Figure 18 shows contrasting images of authentic and counterfeit chips. Since current THz TDS systems are compact, robust and relatively inexpensive, such testing solutions may be readily adapted to large-scale industrial inspection.

A simple approach to testing microwave monolithic integrated circuits (MIMICs) and very large scale integrated (VLSI) circuits were reported by Shur et al. who detected the electrical response generated at the circuit pins when the component was illuminated by THz or sub-THz radiation [119]. They further proposed that machine learning be used to identify characteristic response signatures of circuits having a large number of pins, by evaluating the associated (large) matrix of signal responses. The technique aims to address the problem of hardware testing for performance, authentication and security, and to do so non-destructively, rapidly, and on a large scale.

### 6.2. Solar Cells

THz measurements on solar cells may serve two separate purposes: characterisation and inspection. Characterisation of the solar cell material involves determining its electrical properties in order to predict its performance and efficiency. The parameters measured are typically complex conductivity, charge carrier density and mobility, and their dependence on solar-type illumination. Inspection, on the other hand, involves detecting a variety of flaws and irregularities.

Characterisation of solar cell material has been performed using TDS [120]. However, it lacked sufficient spatial resolution to examine individual cell devices. The LTEM technique (described above) was also employed to image and analyse solar cells [121,122,123]. Of particular interest is the detailed comparison of LTEM with conventional analysis methods, electroluminescence (EL), photoluminescence (PL), and laser beam induced current (LBIC) [123], which found that LTEM is a useful complementary technique that has advantages in spatial resolution and in its ability to observe electric fields, surface defects, grain boundaries, and photocarrier dynamics in the vicinity of the depletion layer.

**Figure 19:**
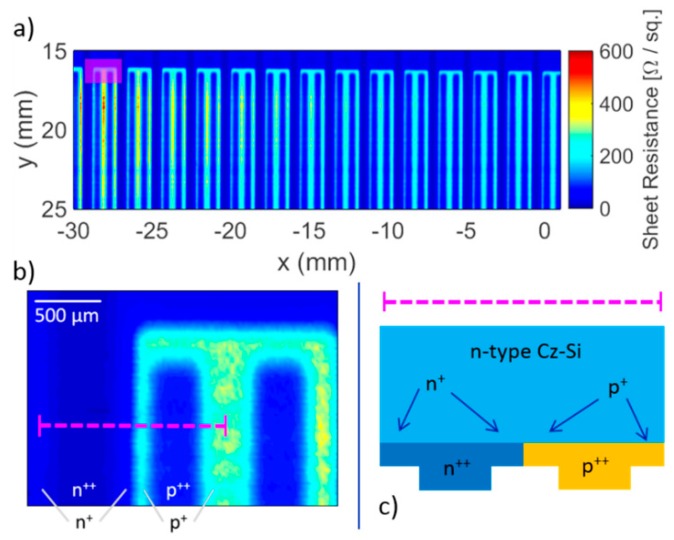
(**a**) A high-resolution sheet resistance map of a solar cell obtained with tip-based THz near-field microscopy. (**b**) Expanded view of a single finger. (**c**) Doping along the line marked in (**b**). Figure courtesy of Protemics GmbH.

TDS and FDS were both employed in reflection mode for inspection of commercial solar cells, successfully detecting manufacturing faults such as defects in tab wires and soldering, cracks, and variations in doping [124,125]. Tip-based near-field transmission measurements improved the spatial resolution of the image down to 10 μm [126,127,128,129,130,131]. The technique produced maps of sheet resistance capable of revealing faults in individual devices (Figure 19).

### 6.3. Graphene

Industrial-scale manufacturing of high-quality graphene sheets is challenging, and the quality of the product depends critically on its conductivity and carrier mobility. Quality and uniformity of graphene sheets are essential for its commercialisation, requiring large-scale inspection techniques that are non-conduct and rapid. THz transmission and reflection measurements can provide solutions where optical techniques cannot. In recent years, THz inspection of graphene has attracted growing interest from both manufacturers and users, and a considerable amount of work has been devoted to mapping conductivity and carrier mobility of graphene sheets, employing techniques similar to those described earlier in this Section [131,132,133,134].

## 7. Petrochemicals

Petrochemicals are particularly suited to the analysis and quality monitoring by THz spectroscopy, because of their high transparency at THz frequencies and the sensitive dependence of their THz dielectric properties on their chemical composition and the presence of contaminants. Although petrochemical products may be classed as low value, their quality control, and condition monitoring can deliver large benefits in cost savings, improved safety, and reduced wastage. Examples include quality control of fuels to improve engine performance and reduce wear, and condition monitoring of lubricating oils to facilitate timely replacement. The petrochemical industry thus appears to be a promising area for THz applications, where much near-industrial work is being carried out, although at present the authors are not aware of any field deployment.

**Figure 20:**
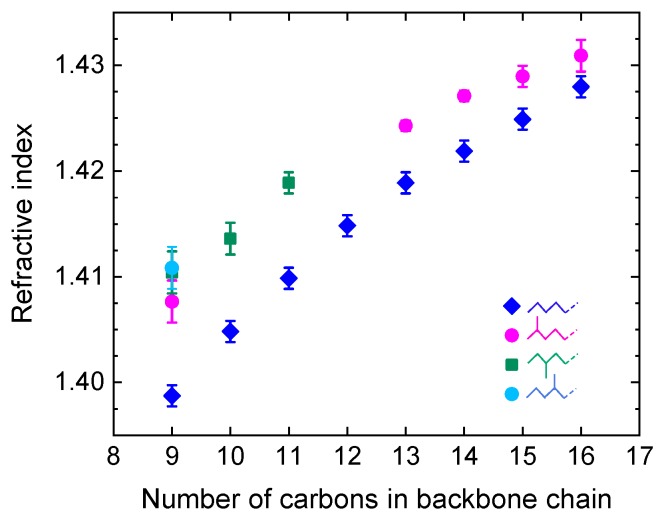
Refractive index at 1 THz (at 20 °C) of alkane compounds with different numbers of carbon atoms in their backbone chain, for both linear and branched compounds, showing the dependence on the carbon number and branching. Adapted from [136].

Petrochemicals consist primarily of hydrocarbon chains of various lengths, mixed with other types of hydrocarbons such as aromatics. They may also contain other flammable components, such as alcohols, as well as non-flammable impurities and contaminants. They are refined from crude oil using distillation processes that separate crude petroleum into grades according to the number of carbons in the molecular chain: gaseous fuels (1–4 carbons), gasoline (5–12 carbons), jet fuel, diesel, heating oil (12–20 carbons), lubricating oils (20–30 carbons), fuel oil (30–40 carbons), and paraffin wax or petroleum jelly (40–50 carbons). Pure hydrocarbons are non-polar substances, which makes them low-loss in the THz range. On the other hand, their THz refractive indices increase monotonically with the number of carbons (Figure 20) [135,136], making it possible to use THz sensing for qualitative and quantitative identification or grading of petrochemicals. This is particularly useful for heavier grades which are opaque in the visible. Moreover, additives or contaminants in petrochemicals (e.g., bioethanol, water, oxidation products) tend to be polar substances that have strong THz absorption, enabling easy detection at low levels of contamination. For all those reasons petrochemicals have long attracted strong interest for industrial implementation of THz sensing, and much literature exists on the subject [137]. Studies have targeted two types of applications: identification and grading; and detection and quantification of contaminants.

### 7.1. Crude Petroleum

THz TDS has been investigated as a tool for identifying the composition of crude petroleum. A combination of THz spectral data and multivariate statistical methods was used to identify crude oils from different oil fields [138]. Other studies looked at oil content in oil shale [139], at the asphaltene content in crude oil [140], at wax crystals in crude oil [141], and at spectral signatures of coal tar [142].

Due to strong THz absorption by water, THz sensing has been employed for measurements of water content in a broad variety of materials, including crude oil (again using TDS). High precision measurements of low water content (0.01–0.25% *w*/*w*) were demonstrated [143], whereas a specially designed sampler was used to measure high water content in the 1.8% to 90.6% range [144]. In a broad and detailed study, oil contamination by both water and solids (e.g., sand) was investigated [145], showing that it is possible to measure concentrations of both. With particular reference to oil fields with a high presence of water, both water content and its distribution were characterized [146].

### 7.2. Fuels

**Figure 21:**
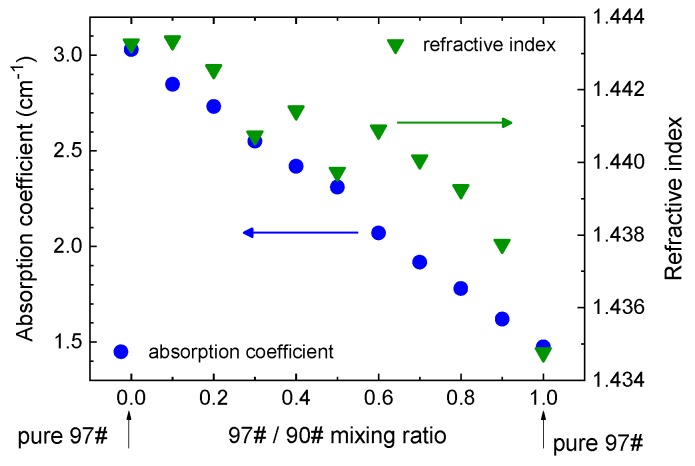
Refractive indices (green symbols, right axis) and absorption coefficients (blue symbols, left axis) at 1 THz of gasoline mixtures of 90# and 97# octane. Adapted from [151].

As in crude oil, THz TDS was applied to identifying fuel grades, additives, and contaminants. A particularly important aspect of fuel analysis is the ability to distinguish and quantify its octane number [147,148,149,150,151,152,153]. This has been demonstrated in several studies: in gasoline and diesel [147], in mixtures of gasoline and diesel [148], in mixtures of 90# and 97# octane gasoline (Figure 21) [149,150], in gasoline of 87#, 89#, and 90# octane [151], and in derv fuel oils [152]. Since fuels with higher octane numbers have shorter carbon chain lengths, their THz refractive indices and absorption coefficients are lower than those of low-octane (longer carbon chains) fuels.

Likewise, it is highly useful to identify and quantify the common additives that are used to augment fuel and to improve its performance [151,153,154]. Evaluation of the cetane number was demonstrated in biodiesel-diesel blends [153], and in ethanol-gasoline mixtures the content of ethanol was determined with 1% accuracy [154,155]. Ethanol being a polar liquid, its addition strongly increases both the refractive index and the absorption coefficient of the mixture compared with those of pure gasoline. Derivatives of benzene (toluene, ethylbenzene, and xylene) commonly comprise a significant volume fraction of gasoline, and these also have been identified using TDS [156]. Concentrations of methyl methacrylate, an additive used to lower the freezing point of diesel, have been detected down to 0.2% [155]. Likewise, sulphur contamination was detected at low concentrations down to 0.2 ppm [155,156]. As with other additives and contaminants that are not pure hydrocarbons, the presence of sulphur even at low concentrations increases THz absorption.

### 7.3. Oils

Lubricating and insulating oils differ from fuels in that their performance depends critically on specific viscoelastic or dielectric properties, and to achieve this they tend to be highly purified and their compositions tightly specified. Moreover, both lubricating and insulating oils degrade in the process of normal operation, therefore, continued performance requires periodic monitoring and replacement. For these reasons, most work in this area has focused on detecting contamination or degradation of oils.

A few studies have used THz TDS for identifying oils [157,158]. In particular, a linear relationship was shown to exist between oil viscosity and its THz refractive index [157], arising from the fact that both viscosity and refractive index increase with the number of carbons in the chain. A related study on contamination of engine oil with gasoline [158] showed that the refractive index of oil decreases and its absorption coefficient increases in the presence of gasoline, with contamination levels down to 4% clearly detectable.

**Figure 22:**
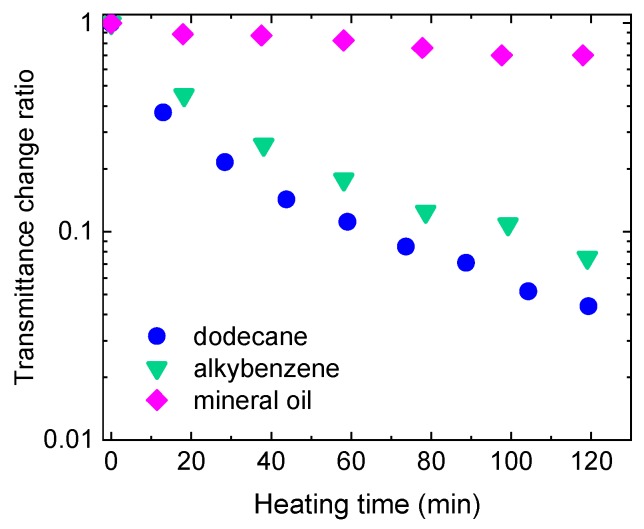
Reduction in THz transmission due to oxidation in three insulating oils. Adapted from [162].

Oils that are used as engine lubricants and as an insulation medium in transformers degrade in operation by processes of oxidation, and there is a need to monitor their condition for purposes of timely replacement. Employing THz sensing to detect oxidation products—carbonyl and hydroxyl compounds—in oils is an effective technique, because those compounds are strongly polar and therefore strongly THz absorbing, allowing detection at low concentrations. A number of studies have been performed demonstrating high sensitivity and good discrimination of such measurements (Figure 22) [159,160,161,162,163,164,165]. Contamination by water can also be a cause of oil degradation, especially in engine oils where water is a product of fuel burning, and where sensitive THz detection has been demonstrated [165,166].

## 8. Gas Sensing

It has long been known that numerous gases have absorption lines in the far-infrared, and a large literature exists on far-IR gas spectroscopy dating back to the beginning of the last century, with studies focusing on gases of interest to astronomy, astrophysics, and atmospheric science. From the early days of THz instrumentation, it was realised that THz measurements can contribute useful data to this area, leading to an extensive body of work. Since gas absorption lines are commonly very narrow—a few GHz at atmospheric pressure (linearly proportional to pressure)—and their central frequencies have been previously established or can be calculated, many of these studies used narrow-band high-resolution techniques, often employing electronic devices, in contrast to the broadband photonic TDS typically employed in other areas of THz applications. Although electronic systems are incapable of delivering frequencies and bandwidths as high as photonic systems, target gas species have sufficient lines at lower frequencies and in narrow bands to permit detection and identification.

Many of the examined gases are of interest in pollution monitoring and Earth observation. However, in the early days of THz measurements, it was not feasible to observe gases in the environment. With the recent development of THz technologies, it became possible to perform measurements in the field and to detect various gases in the ambient atmosphere. This led to a resurgence of interest in applications-focused THz gas detection and monitoring, and the growing awareness of its advantages. Many of the gases of interest do not have lines in the near-IR, where sensor technologies are most effective and available. In contrast, in the mid-IR where the great majority of gases have their main absorption bands, spectroscopy is challenging, requiring larger and more expensive sensors than in the near-IR. Moreover, polyatomic gases have dense and complex absorption bands in the mid-IR with a high multiplicity of lines, making it difficult to achieve sufficient specificity and to identify gas mixtures. At THz frequencies, gases have relatively few lines, facilitating specificity and selectivity, and the current technology is able to provide requisite detection sensitivity while also being sufficiently robust and compact. Three areas have attracted particular interest: environmental monitoring, including pollutants and Earth observation, human breath analysis, and quality monitoring of natural gas in the production and supply chain.

### 8.1. Environmental Monitoring

The area of gas sensing for environmental monitoring includes detection of pollutants and contaminants to meet the requirements of health and safety and monitoring of atmospheric components for weather and climate observation. Typically, detection of pollutants demands high sensitivity, selectivity and specificity; whereas atmospheric monitoring may have less stringent requirements.

Commercial terahertz spectrometers for trace gas sensing were already developed a decade ago, employing precisely tunable diode lasers and photomixers (see FDS in Section 1) [167].

A recent review of toxic compound detection by THz spectroscopy by Yang et al. [168] lists a wide range of gases studied. In a work focused on demonstrating selectivity, TDS was used to examine eight gases [169]: acetaldehyde, acetonitrile, ethanol, water, methanol, ammonia, propionaldehyde, and propionitrile. In contrast, focusing on sensitivity and also using TDS, detection at concentrations of less than 10 ppm was achieved in carbon monoxide, methanol, water, and acetonitrile [170].

Much work in this area has been devoted to developing dedicated electronic systems for gas sensing, comprising both a source detector assembly and a gas cell for collecting and concentrating gas samples. The advantage of all-electronic systems for this task is their compactness, robustness and low power consumption. Their narrow frequency range can be tolerated provided that it is tailored to the specific gases examined. In an early work, methyl fluoride, difluoromethane, carbonyl sulphide and methyl iodide were detected in the range between 254–256 GHz [171].

**Figure 23:**
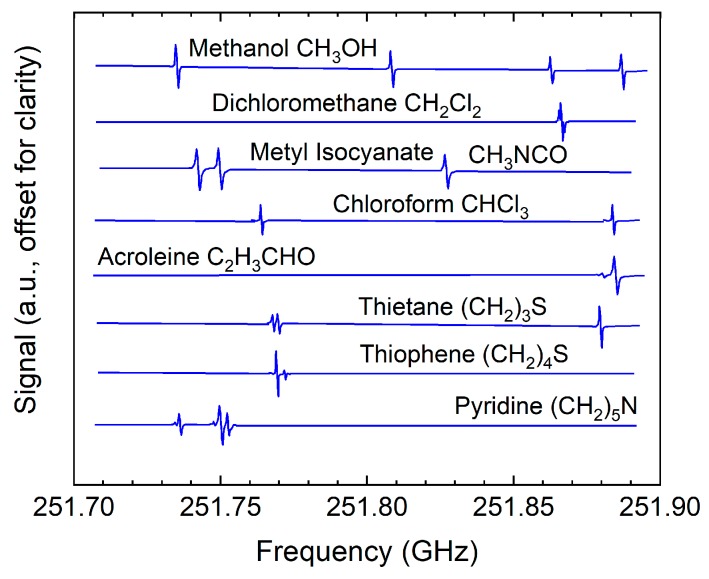
Expanded section of the detected spectrum showing spectral signatures of eight gases. Adapted from [172].

By building a purpose-designed system that comprised an electronic sensor operating between 210–270 GHz and a gas cell capable of collecting and concentrating gas samples, Neese et al [172] were able to detect and identify mixtures of 14 gases (acetonitrile, methyl fluoride, acrylonitrile, sulfur dioxide, methyl iodide, methyl bromide, trifluoromethane, acrolein, propionitrile, 1,1 difluoroethene, vinyl fluoride, oxetane, vinyl bromide, 1,2 dichloroethane) at ppt levels, meeting the requirements of the Clean Air Act (http://www.epa.gov) (Figure 23). Similar custom-built gas sensing electronic systems were described in [173,174]. Another custom-built sensing system and the gas cell were used to monitor food spoilage by detecting hydrogen sulphide at around 611.4 GHz, achieving a sensitivity of 0.5 ppm [175].

In contrast, a system employing a vector network analyser (VNA) at 238–252 GHz was used to detect acetaldehyde, methanol, deuterated methanol, acetone, isopropyl alcohol, acetonitrile, and ethanol, and their mixtures [176]. Although good specificity and selectivity were demonstrated, such a VNA-based system is less suited to field deployment due to its lack of compactness and robustness and its high cost.

In addition to toxic gases, detection of particulates in the atmosphere is also vitally important for pollution monitoring. Two studies have used TDS to measure levels of PM2.5, i.e., concentration of particles with a mean size of 2.5 μm [177,178], detecting concentrations as low as 20 μg/m^3^.

High-resolution THz instruments may also be employed as remote toxic gas sensors at disaster sites. An FDS system and a multipass cell evacuated to 1 mbar were used to detect trace amounts of gases that might be released in a blaze or a chemical spill in an industrial facility, achieving detection limits in the range of 20 ppm for ammonia (NH_3_) and 100 ppm for sulfur dioxide (SO_2_) [179]. The fact that terahertz radiation is able to penetrate optically opaque environments, such as black smoke, was exploited to identify hydrogen cyanide (HCN) and water molecules generated by the combustion of a urethane foam block [180].

### 8.2. Breath Analysis

Analysing gases exhaled in human breath is a highly promising application, with the potential for early detection of disease biomarkers. Such detection would be fast and non-invasive, facilitating early diagnosis and state-of-health monitoring. Potential detectable diseases include lung cancer, diabetes, some neurological disorders, and smoking and alcohol consumption.

**Figure 24:**
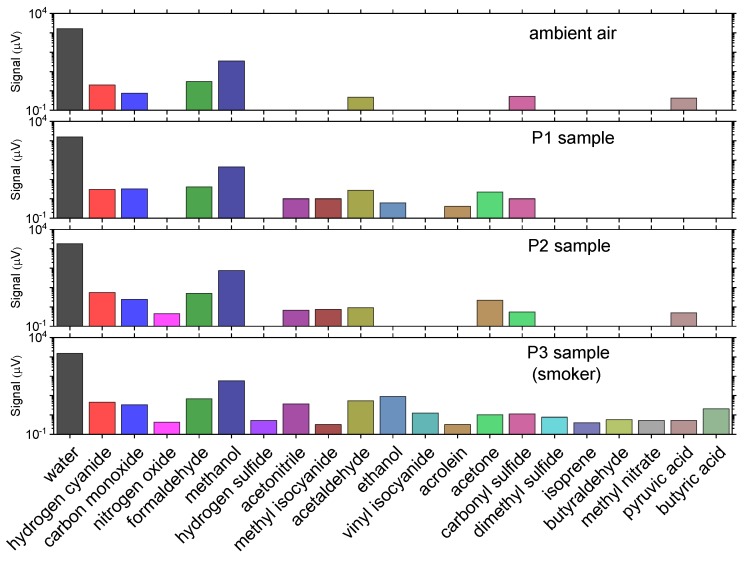
Gases detected in exhaled human breath. Adapted from [183].

Custom-designed electronic systems were used to detect alcohol [181] and several bio-marker gases [182] in breath with good sensitivity. Rothbart et al. [183] employed a specially designed gas cell which the probe beam would traverse multiple times (11 passes) to extend the interaction length with the contained gas and thus increase the absorption signal. Measuring between 220–330 GHz, they were able to detect 21 gases (water, hydrogen cyanide, carbon monoxide, nitrogen oxide, formaldehyde, methanol, hydrogen sulfide, acetonitrile, methyl isocyanide, acetaldehyde, ethanol, vynil isocyanide, acrolein, acetone, carbonyl sulfide, dimethyl sulfide, isoprene, butyraldehyde, methyl nitrite, pyruvic acid, butyric acid) (Figure 24). Moreover, they observed a clear distinction between smoker and non-smoker samples.

### 8.3. Natural Gas

Natural gas is primarily a mixture of three hydrocarbons: methane (CH_4_), ethane (C_2_H_6_), and propane (C_3_H_8_), the ratio of which determines its calorific content, i.e., its quality as fuel. In addition, it usually contains an admixture of some carbon monoxide (CO) and carbon dioxide (CO_2_), which are contaminants. Therefore quality monitoring of natural gas is of some importance. Unlike pollution and breath monitoring, in this application high sensitivity, selectivity and specificity are not required.

**Figure 25:**
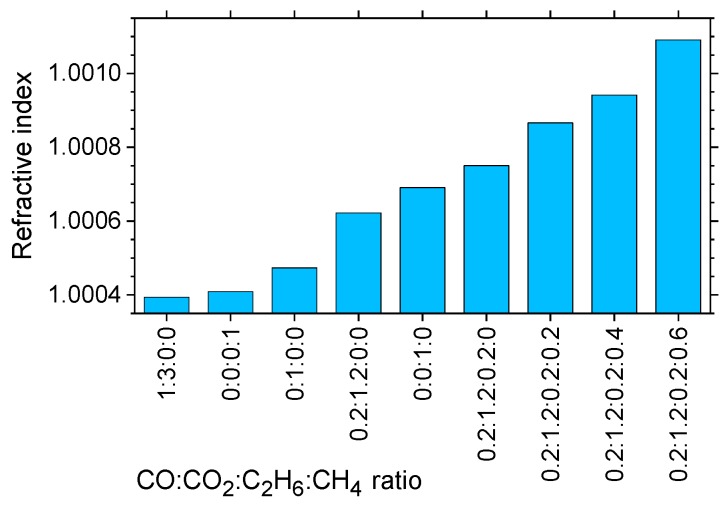
Refractive indices (mean over 0.3–1.3 THz) of natural gas constituents and their different mixes. Adapted from [184].

TDS was used to demonstrate that THz measurements can establish the mixing ratios of natural gas [184,185]. As seen in Figure 25, the refractive index provides a good indicator of the mix, due to the differences in the indices of individual gases.

## 9. Paper and Wood

### 9.1. Paper

Although all types of paper have high THz absorption, thin paper sheets have moderate-to-good THz transparency. THz absorption by the paper is determined by its thickness, composition, moisture content and texture, allowing these properties to be evaluated using THz transmission measurements. THz sensing, therefore, offers a solution to the paper industry’s need for in-line monitoring of paper caliper (thickness), moisture content and basis weight (weight per area). As a result, considerable amount of work has been devoted to investigating the feasibility of this application, including several industrial demonstrations.

**Figure 26:**
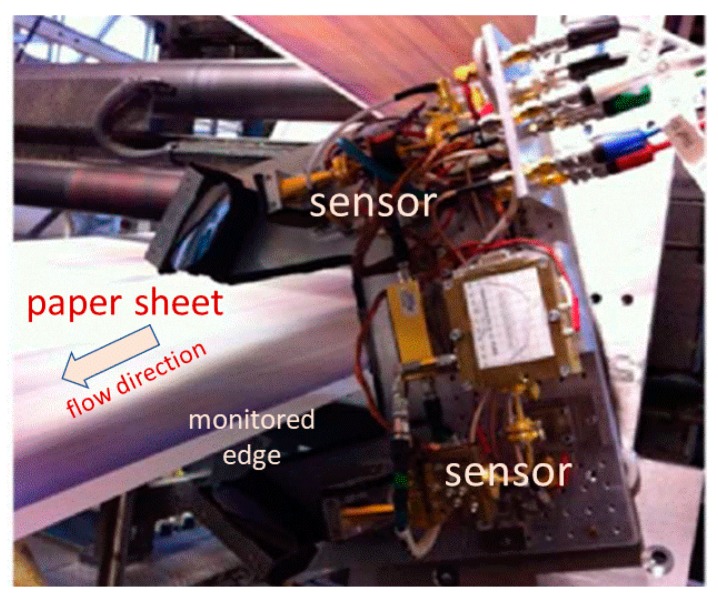
An industrial prototype of an electronic sensor at 200 GHz monitoring paper moisture during offset printing. Adapted from [197].

Laboratory studies demonstrated diagnosing moisture content [186], simultaneous measurements of thickness and moisture [187,188], and multiple parameter determination [189,190,191,192,193]. Aiming at a more compact system, a THz fiber Bragg grating was used to measure paper thickness in [194]. The area was partially reviewed in [195]. Monitoring the ageing of oil-paper insulation in transformers was investigated in [196]. Vassilev et al. used a purpose-built electronic system at 200 GHz to monitor moisture levels in paper during an offset printing process, demonstrating an industrial prototype (Figure 26) [197].

**Figure 27:**
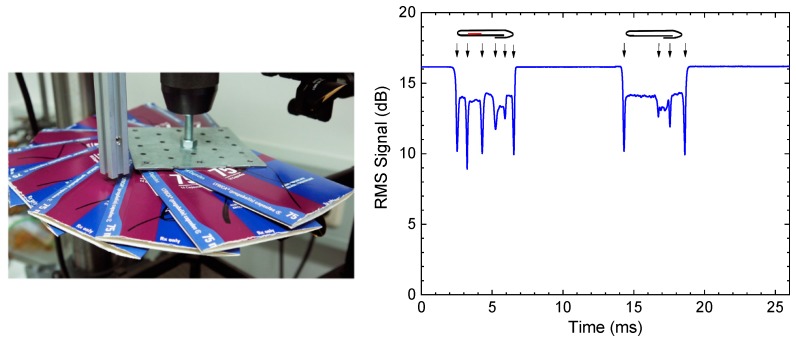
(**a**) Boxes arranged on a fast turntable. (**b**) 1D-scan of folded cardboard boxes with and without a package slip (shown in red). The graph depicts the transmitted THz intensity with the boxes moving at 21 m/s. Adapted from [198].

In a paper-related industrial demonstration, Brinkmann et al. used TDS for quality control of cardboard boxes as used in pharmaceutical packaging [198]. Their setup combined a pulsed THz emitter with a high-bandwidth Schottky receiver and accomplished THz intensity measurements with an effective time resolution of 6.4 µs. In a proof-of-principle experiment, they showed that THz screening was able to detect the presence or absence of package inserts inside the cardboard boxes unambiguously, even for samples moving at more than 20 m per s (Figure 27).

### 9.2. Wood

**Figure 28:**
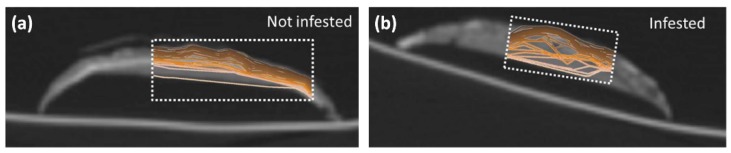
THz scans overlaid on CT images, revealing beetle damage. a) Healthy wood; b) infested wood. Reprinted with permission from [205] © The Optical Society.

Wood has also been investigated with THz spectroscopy, although it is less transparent to THz waves than paper due in part to the larger thickness of samples and a stronger contribution from scattering. Due to its anisotropic structure consisting of aligned fibers, wood is birefringent, making it possible to detect orientation [199,200,201]. Similar to paper, moisture content and density of wood have also been measured [202,203]. Krügener et al. studied THz optical properties of several species of wood with a view to identifying and evaluating wood samples [204]. They also used a THz reflection system with a robotic arm to scan wood logs in order to reveal areas underneath the bark that had been damaged by burrowing “typographer” beetles (Figure 28) [205].

## 10. Conclusions

The often-quoted phrase “THz moves out of the lab” finally seems to be coming true. The authors still remember panel discussions at THz conferences about a decade ago, when none of the attending experts would have been able to name but a single “killer application”. Today, however, THz technologies show a significant market potential, a finding that is confirmed by a number of recent market reports [206,207,208,209,210]. Admittedly, the term “killer application” remains questionable, yet applications especially in the field of non-destructive, non-contact testing now successfully exploit the abilities of THz radiation, i.e., penetration of many opaque materials, depth resolution on the micrometer level, high spatial resolution as compared to microwaves, to name but a few. Industrial applications are made possible by the availability of more advanced, yet robust and long-lived systems, and benefit from more competition between manufacturers, which has led to a significant price drop for THz instrumentation. Whilst Hochrein wrote in 2014, “The average system price is approx. 134,000 EUR” [6], most of today’s TDS “base systems” cost about 1.5 to 2 times less. From the perspective of an industrial user, inexpensive systems amortize faster than costly ones, and perhaps for the first time in the history of THz technologies, an investment in THz instrumentation results in actual cost *savings*: thinner walls of plastic pipes or bottles reduce material usage and wastage (Section 3); a fully automated, robot-driven system for paint layer measurements reduces production times in the automotive industry (Section 4); an optimum tablet coating may improve the efficacy of a drug and therefore increase its chance of success in clinical trials (Section 5); an early identification of faults in electronic structures enhances the yield and helps reduce failures in the field (Section 6). These examples are not just hypothetical but are based on industrial installations that the authors are aware of. Readers may refer to the websites of system manufacturers as listed in the references of Section 3, Section 4, Section 5 and Section 6—the most advanced markets at the time of writing—for further details.

For other industries, including paper, wood, but also petrochemicals, numerous publications have shown the technological potential of THz instrumentation, but apart from initial tests, market acceptance has been slow—either because alternative and established methods exist, or because markets are more price-sensitive. In fact, any THz system manufacturer will have received customer inquiries such as “I need a THz sensor, but it must not cost more than 10,000 EUR”, and this price level remains out of reach today. This may change in the future, e.g., with miniaturized on-chip systems, but a large amount of work remains to be done, which still creates something of a “chicken-and-egg” problem. In the special case of gases, a commercial instrument was developed more than 10 years ago [167], but for reasons not known to the authors, the project was abandoned and sales seem to have been discontinued.

**Figure 29:**
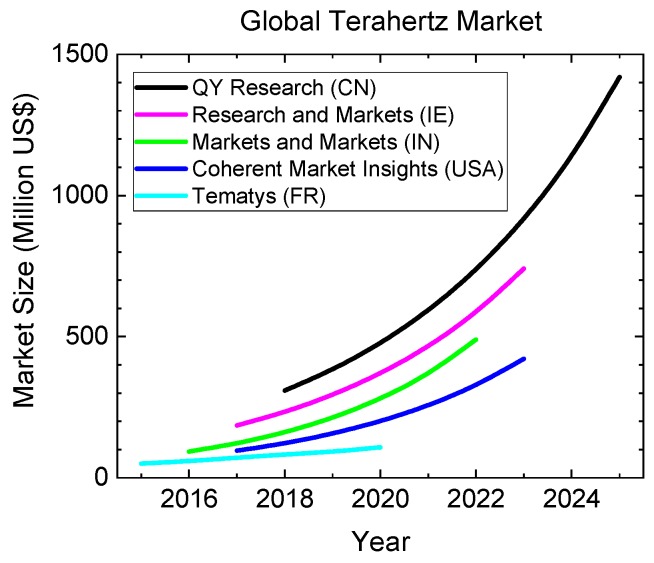
Global terahertz market in Million USD, according to five different analyst firms [206,207,208,209,210].

The total THz market volume seems difficult to quantify. It is noteworthy that the aforementioned market reports differ in their estimate for 2019 by more than a factor of 4 (Figure 29). According to Tematys, a French research company, the total market is still significantly below 100 Million US$ [209]. On the other end of the spectrum are Beijing-based QY Research, who believe in present market size of 385 Million US$ [206]. Similarly, predictions for the compound annual growth rate (CAGR) differ by a factor of two, with values ranging from 16% (Tematys) to almost 32% (QY Research). Taking a simple arithmetic average of the five reports, the global market amounts to approximately 230 Million US$ and the CAGR is roughly 25%.

Nonetheless, all of the reports agree that the market for THz technologies will continue to expand, and even the most “pessimistic” prediction of a CAGR of 16% still indicates a significant growth. It remains to be seen which applications will have won the broadest industry acceptance by the time of the next review.
